# Breast cancer and pregnancy: a comparative analysis of a Chilean cohort

**DOI:** 10.3332/ecancer.2014.434

**Published:** 2014-06-03

**Authors:** César Sánchez, Francisco Acevedo, Lidia Medina, Carolina Ibáñez, Dravna Razmilic, M. Elena Navarro, Mauricio Camus

**Affiliations:** 1 Haematology–Oncology Department, Cancer Programme, School of Medicine, Pontificia Universidad Católica de Chile, Diagonal Paraguay 319, Santiago 8330032, Chile; 2 Radiology Department, Cancer Programme, School of Medicine, Pontificia Universidad Católica de Chile, Diagonal Paraguay 319, Santiago 8330032, Chile; 3 Oncology–Surgery Department, Cancer Programme, School of Medicine, Pontificia Universidad Católica de Chile, Diagonal Paraguay 319, Santiago 8330032, Chile

**Keywords:** breast cancer, pregnancy, prognosis

## Abstract

**Introduction:**

Recent reports show that pregnancy-associated breast cancer (PABC) survival is similar to that of non-pregnant young patients. We evaluate the characteristics and prognosis of PABC patients treated in our cancer centre.

**Patients and methods:**

We identified patients with invasive PABC who were treated between 1999 and May 2013 and compared their characteristics with a no PABC cohort of similar age.

**Results:**

The prevalence of PABC was 1% (*n* = 17). The median age was 35 years (range: 29– 42 years). The initial tumour was suspected clinically in 93% of the cases. Total mastectomy rates were higher in women with PABC (78.6% versus 40.5%, *p* = 0.02), and more tumours in the PABC group were triple negative, epidermal growth factor type 2 (HER2)–positive, and at advanced stages; however, these differences were not statistically significant. While estimated overall survival at ten years was higher in the non-PABC group (75.5% versus 80.5%, *p* = 0.043), disease-specific survival (DSS) rate at ten years was not statistically different between groups (83.9% for PABC and 75.5% for unrelated pregnancy BC, *p* = 0.37).

**Conclusions:**

PABC is a rare event. In our cohort, it tended to be more aggressive. Compared with a similar age cohort, the DSS was not worse.

## Introduction

The frequency of cancer during pregnancy is estimated at 1 in 1000 women. Breast cancer, melanoma, and cervical tumours are the most common neoplasms associated with pregnancy, followed by haematological neoplasm [1].

Because there had been a trend towards later childbearing in the past four decades, added to the fact that cancer incidence increases with age, more women are being diagnosed with cancer during the course of pregnancy or within a short time after delivery; however, the diagnosis of cancer during pregnancy is still an uncommon event [1, 2].

Breast cancer is the leading cause of death due to cancer in Chilean women [3]. In our centre, the median age of women affected by breast cancer is 55 years, and 12% of them are under 40 years at diagnosis, and then a large percentage of our patients are of childbearing age (data not published).

Pregnancy-associated breast cancer (PABC) has traditionally been associated with a worse prognosis and overall survival [2, 4, 5]. However, two recent publications analysing the prognosis of PABC and pregnancy after the treatment of a breast cancer do not show an unfavourable outcome when comparing this group with patients of the same age [6, 7].

Our goal is to evaluate the clinical presentation and prognosis of women with PABC, defined as breast cancer presented during pregnancy or up to one year after delivery, and compare them with those of a cohort of women of the same age with breast cancer not associated with pregnancy, treated at the same institution.

## Patients and methods

In this descriptive study, we identified patients with invasive PABC, treated in the Cancer Centre of the Pontificia Universidad Católica de Chile, Santiago, Chile, between January 1999 and May 2013. We define PABC as the breast cancer presented during pregnancy or up to one year after delivery. We recorded age, reason for consultation, timing of diagnosis in relation to pregnancy, type of surgery (total versus partial mastectomy), use of systemic therapy, tumour pathological findings, overall survival (OS), and disease-specific survival (DSS). OS was defined as the time from the initial diagnostic biopsy until death from any cause. The date and cause of death data were obtained from death certificates obtained from the civil registration records, and from our clinical database. Pathological analysis included histological type and differentiation grade as defined by Elston and Bloom and modified by Elston and Ellis [8]; study of ER and progesterone receptor (PR), range 0–100%; and HER2 scale + to +++. All methods through immunohistochemistry. HER2 scale may be defined as follows: +, negative; ++, uncertain and further evaluated by immunofluorescence *in situ* hybridization (FISH), and +++, positive. Tumour clinic pathological subtype was defined according to the following: Luminal A (ER+ and/or PR+, histological grade (GH) 1–2, and HER2 negative), luminal B (ER+ and/or PR+, GH 3, and/or HER2 positive), triple negative (TN) (ER−, PR−, HER2 negative), HER2+ (ER− and PR−, and HER2 positive) [9]. DSS was defined for the same period as OS, considering only patients who have died of breast cancer.

We compared the findings of PABC patients with that of a cohort of patients with breast cancer not related to pregnancy, in the same age range and treated at our institution during the same period. Data were analysed with SPSS 21.0 software using descriptive statistics. Survival curves were calculated according to the Kaplan–Meier analysis and compared with log-rank test. *p* < 0.05 was considered statistically different.

## Results and discussion

### Results

**Figure 1.**
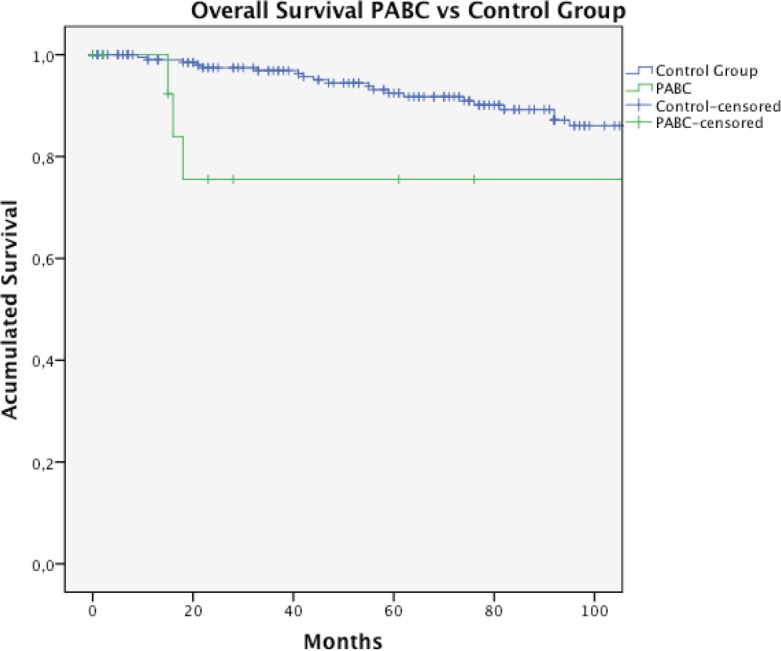
Overall survival in PABC and unrelated pregnancy breast cancer control group.

**Figure 2.**
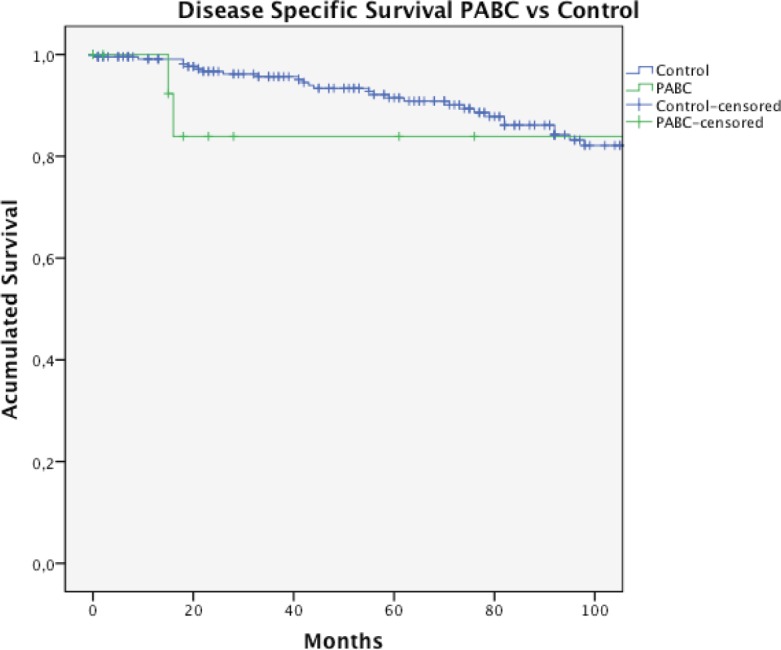
DSS rate in PABC and unrelated pregnancy breast cancer control group.

We recorded 1631 patients with breast cancer in stages I–IV during the period. Of these, 17 correspond to PABC, with a prevalence for this last presentation of 1%. In the case of two patients, we did not get enough data for this study. The characteristics of the remaining patients (15) are described in Tables 1 and 2. The median age was 35 years and the initial suspected diagnosis of the tumour was clinical in 93% (14/15) of cases. Most patients were diagnosed during pregnancy (53.3%). The most common histological type was invasive ductal carcinoma in 86.6% of cases, and 66.6% were ER positive. According to our definition of clinic pathological subtypes (Materials and methods section), histological subtype luminal (66.6% of cases) was distributed in 3/14 LA and 4/15 LB, and the other 3/14 luminal was not classifiable because of lack of data. Axillary node compromise was found in 66.6% of the cases. Fifty-three per cent of patients are diagnosed at stage III. Five patients received chemotherapy during pregnancy. Seventy-one per cent of patients were managed with total mastectomy. We did not obtain surgery information for one patient who received neoadjuvant therapy and underwent surgery in another centre. The prevalence of PABC in women with breast cancer diagnosed between 29 and 42 years was 6.3% (17/268). When comparing both groups, we noted that women with PABC were treated more frequently with total mastectomy (78.6% versus 40.5%, *p* = 0.02), presented with tumours in more advance stages, and presented with a more aggressive phenotype (higher frequency of stage III, TN, and HER2-positive tumours, see Table 1); however, the phenotype differences were not statistically significant. With a median follow-up for the total group of 71.5 months (1–173 months), the estimated OS of women with BCAP at ten years was 75.5% compared to 80.5% in the control group, *p* = 0.043 (Table 3 and Figure 1). Four patients with PABC have died: three of them because of breast cancer. Two patients suffered a recurrence of the disease. The DSS at ten years was higher for PABC women, but not statistically different between both groups (Table 3, Figure 2).

### Discussion

Breast cancer is one of the most frequently encountered malignancies during pregnancy. About 0.2–2.6% of breast cancer occurs during pregnancy [1], a similar figure to that found in our cohort.

The delay in the age at first pregnancy explains why the frequency of PABC has increased. In countries like Sweden, the incidence of PABC has grown from 16/100,000 births in 1963 to 37.4 per 100,000 in 2012 [4].

Because the main risk factor for the development of breast cancer is exposure to oestrogen, it has been theorised that pregnancy can stimulate and aggravate the evolution of breast cancer [1]. The diagnosis may be difficult because the anatomic changes of the breast are characterised by a presentation of the disease in later stages, with poorly differentiated tumours, often ER negative [1, 4]. The delay in diagnosis is probably explained by the difficulties in the physical examination and the general assumption that a tumour in the breast is benign and possibly related to pregnancy. The worst prognosis of PABC has been described primarily in retrospective studies [10]. Differences in case’s definition, inclusion of more advanced disease, and delayed initiation of treatment may influence the unfavourable results of those series [2, 10]. Most of these studies, given its retrospective nature, contain no systematic detail of the tumour’s characteristics, and it is difficult to determine how to apply these results to clinical practice [10].

Knowledge of the disease and the experience gained in its management during pregnancy have allowed the development of consensus management, diagnosis, and treatment of PABC [1, 2]. A breast mass during pregnancy that is not resolved in two weeks should be studied. The staging of the disease with mammography and radiation protection, abdominal ultrasonography, and magnetic resonance without gadolinium has been proven to be safe during pregnancy. Further treatment of the disease during pregnancy, with surgery and chemotherapy, is effective and feasible [1].

The use of chemotherapy after the first trimester has been widely reported based on schemes such as fluorouracil, adriamycin, and cyclophosphamide [1, 4], which has been used in our group [11], and largely on the experience of others, not only in breast neoplasms [1, 12].

Current studies suggest that in breast cancer during pregnancy receiving standard regimens and treatment, there does not appear to be any significant difference in survival when compared with similar control groups. Amant *et al* [6] collected data from cancer registries and cooperative multinational studies, and analysed the prognosis of 311 women with breast cancer, who were diagnosed and treated during pregnancy. Patients diagnosed with breast cancer postpartum were excluded from this analysis. Interestingly, no difference in mortality between this group and a cohort of the same age with breast cancer unrelated to pregnancy was found. These findings suggest that pregnancy has no negative impact on breast cancer, giving relevant information for counselling women who are diagnosed during pregnancy [10]. However, the same conclusion is not extended for patients with breast cancer diagnosed in the postpartum period. In this situation, the prognosis seems to be worse [13, 14]. We included both populations (breast cancer diagnosed during pregnancy and after one year of delivery) to increase the number of patients.

Our work is aimed at analysing our experience and evaluating the prognosis of these patients compared with other young women with breast cancer not associated with pregnancy treated in a single institution. The limitations of our study are mainly based on the retrospective nature of the data and the small sample size. However, it emphasises the experience of an institution, compared with a control group of the same age. As reported in other studies, most tumours are diagnosed by symptoms or signs, not through screening studies, not surprisingly in patients under 40 years; most of the tumours are diagnosed in locally advanced stages (>50% in stage III), with higher frequency of nodal involvement, less ER, and high HER2 overexpression. Interestingly, compared with a local cohort group, PABC patients tend to have shorter overall survival, but the specific mortality cause is no different between groups.

## Conclusions

The association of breast cancer and pregnancy is becoming more common. Recent studies show that the diagnosis of breast cancer during pregnancy does not seem to adversely affect the prognosis, when they are compared to the same extent with patients of the same age. Our results show that the clinico-pathological features of PABC tend to be more aggressive compared to an aged-matched control group; however, these characteristics do not imply worse DSS. To improve our data, we are prospectively recording the information of our patients with cancer in relation to pregnancy.

## Figures and Tables

**Table 1.** Clinic pathologic characteristics of 15 patients with PABC, compared with 251 women of same age range, with breast cancer unrelated to pregnancy.

	PABC		Control group		*P* value
	*N*: 15	%	*N*: 251	%	
Age median (range)	35.3(29–42)		38(29–42)		
Histological type					
Ductal	13/15	86.6	203/251	80.9	0.57
Others	2/15	13.3	48/251	19.1	
Node involvement	10/15	64.2	129/233	55.4	0.39
Total mastectomy	11/14#	78.6	94/232	40.5	0.02*
Axillary dissection	9/13#	69.2	155/229	67.7	0.96
Stages					
I	2/15	13.3	72/238	30.3	0.16
II	5/15	33.3	97/238	40.8	0.57
III	7/15	46.7	62/238	26.1	0.08
IV	1/15	6.7	7/238	2.9	0.42
Subtypes					
ER/PR positive	10/15	66.7	186/240	77.5	0.33
HER2 positive*	2/15	13.3	12/236	5.1	0.17
Triple negative	3/15	20.0	37/236	15.6	0.65

PABC: pregnancy-associated breast cancer; ER: estrogen receptor; PR: progesterone receptor;

HER2: epidermal growth factor receptor.

* HER 2 enrich.

# Patients with enough clinical data.

**Table 2.** Characteristics of 15 patients with PABC. Relation between delivery and time to diagnostic.

	Number	%
Diagnostic during pregnancy		
First trimester	3/15	20
Second trimester	4/15	26.6
Third trimester	1/15	6.6
After delivery	7/15	46.6
Time to diagnosis after delivery (months)	2.75 (0.5–4)	

**Table 3.** Overall survival and DSS rate of 15 patients with PABC, compared with 251 women of same age range, with breast cancer unrelated to pregnancy.

Estimated survival at ten years	%	%	*P* value
	PABC	Control group	
OS	75.5% (±12.3)	80.5 (±3.9)	0.043*
DSS	83.9 (±10.4)	75.5 (±4.2)	0.37

* Statistically significant; OS: overall survival; DSS: disease-specific survival; PABC: pregnancy-associated breast cancer.
